# A single session online training reduces intolerance of uncertainty and improves mental health in emerging adults

**DOI:** 10.1017/S0033291725102419

**Published:** 2025-12-15

**Authors:** Sarah Daniels, Yasmin Hasan, Susanne Schweizer

**Affiliations:** School of Psychology, University of New South Wales, Sydney, Australia

**Keywords:** adolescence, anxiety, depression, digital training, intolerance of uncertainty, transdiagnostic

## Abstract

**Background:**

High uncertainty in recent global health, geopolitical, and climate crises has been proposed as one important driver of the rise in youth mental health problems. This makes intolerance of uncertainty – a transdiagnostic risk factor for mental health problems – a promising target for intervention.

**Methods:**

This study presents a novel single-session online training that took a synergistic mindset approach to promote uncertainty-as-adaptive and growth mindsets. The novel Uncertainty-Mindset Training was compared with Psychoeducation and No-Training control groups in 259 older adolescents/emerging adults (18-to-24-year-olds).

**Results:**

The Uncertainty-Mindset Training reduced intolerance of uncertainty, anxiety symptoms, and depression symptoms 1 month later. Importantly, the clinical gains were mediated by reductions in intolerance of uncertainty.

**Conclusions:**

Given that this ultra-brief training can be delivered at scale globally and at no cost to the users, it shows promise for significant public health impacts.

## Introduction

From wondering about when and where the next natural disaster will strike, to living through a global cost-of-living crisis and pandemic, adolescents today are coming of age amidst great climate, economic, social, and health uncertainty. High uncertainty has been proposed as a driver of the rising rates of youth mental health problems (Schweizer, Lawson, & Blakemore, 2023; The Lancet, 2022), especially for individuals high in intolerance of uncertainty (IU). IU, reacting more negatively to uncertainty (Freeston, Rhéaume, Letarte, Dugas, & Ladouceur, 1994), is a transdiagnostic risk factor for mental health problems including depression and anxiety (Mahoney & McEvoy, 2012; Morriss, 2025). Reducing IU through easily accessible interventions then has the potential for significant impact on youth mental health.

Adolescents and emerging adults experience substantial uncertainty in their daily lives (e.g. rapidly changing relationships and career decisions; Arnett, Žukauskienė, & Sugimura, 2014; Blakemore, 2019). Compared to adults, adolescents have a greater willingness to engage in, and are more successful in, the behavioral exploration of uncertain environments (Lloyd, McKay, Sebastian, & Balsters, 2021); yet, this increased engagement with uncertainty appears to come at a cost to their emotional well-being (Schweizer et al., 2023). Compared to adults and older adults, adolescents and emerging adults report higher IU (Okayama et al., 2024), which is associated with poor mental health (Frank et al., 2012; Gentes & Ruscio, 2011). Consequently, while adolescents are more likely to engage with uncertainty, they tend to find this uncertainty aversive and may thus experience greater psychological distress. Reducing IU is therefore a promising target to promote youth mental health.

Meta-analyses indicate that a range of interventions can improve IU with large effects as compared to control interventions and waitlist groups (*g* = 0.89, Miller & McGuire, 2023; *g* = 1.35, Wilson, Abbott, & Norton, 2023). Cognitive behavior therapy is one such approach which has been shown to substantially reduce IU, leading to less anxiety (Zemestani, Beheshti, Rezaei, van der Heiden, & Kendall, 2021) and improved functioning (Palitz et al., 2019). While clinician-led cognitive behavior therapy effectively improves IU, several barriers including stigma, time, and financial costs prevent access to, and engagement with, evidence-based care (Brown, Rice, Rickwood, & Parker, 2016; van den Heuvel, Barozzino, Milligan, Ford-Jones, & Freeman, 2016), with two-thirds of diagnosed youth not receiving the help they need (Merikangas et al., 2011). To help facilitate access to mental health support, several online cognitive-behavioral therapy-based interventions have been developed that reduce IU in individuals with anxiety disorders (Bouchard et al., 2022; Hedman et al., 2013). While advances in digital health interventions are improving global access to mental health care, stigma and substantial time commitments are not addressed by simply moving interventions to online platforms and apps. To halt the tide of the global youth mental health crisis, fundamental innovation in prevention and intervention approaches is needed. Here, we combined two such developments: 1) improving mental health indirectly by targeting IU, an underlying mechanism that does not have the same associated stigma as mental health (Cuijpers & Reynolds, 2022; Smout et al., 2024), and 2) adopting a Single-Session Online Intervention (SSOI) approach (Schleider & Weisz, 2017), which takes away the burden on individuals’ most precious commodity: time, thereby facilitating access.

SSOIs are low/no-cost, self-guided, easy-to-disseminate digital interventions for mental well-being, making them inherently scalable (Schleider, Dobias, Sung, & Mullarkey, 2020). SSOIs demonstrate comparable efficacy to therapist-administered single-session interventions, which show small-moderate effects transdiagnostically (Schleider & Weisz, 2017). Additionally, SSOIs overcome the issue of attrition, with multisession online interventions showing attrition rates as high as 99% (Zhou, Edirippulige, Bai, & Bambling, 2021). To potentiate their impact, SSOIs must target mechanisms involved in a wide range of disorders and be transferable to individuals’ unique and diverse experiences. Translating these core tenets of SSOIs, we developed a single-session online IU-focused training including three components: 1) promoting an uncertainty-as-adaptive mindset, 2) an uncertainty tolerance growth mindset, and 3) repetitive negative thinking cessation.

The rationale for promoting *uncertainty-as-adaptive mindsets* is that individuals with high IU tend to have an uncertainty-as-debilitating mindset, believing that uncertainty is inherently negative and a threat (Dugas et al., 2005; Reuman, Jacoby, Fabricant, Herring, & Abramowitz, 2015). One SSOI, which highlighted uncertainty as unavoidable and nonthreatening, significantly reduced emerging adults’ IU one month later, with change in IU partially mediating the intervention’s effects on anxiety and depression (Shapiro, Allan, Raines, & Schmidt, 2023). Appraising uncertainty as potentially beneficial is associated with better physical and psychological quality of life (Padilla, Mishel, & Grant, 1992). The current training therefore explicitly promoted benefits of facing uncertainty.

The training was based on a synergistic approach: combining the uncertainty-as-adaptive module with a *growth mindset* module, highlighting that all individuals have the capacity to improve their ability to cope with uncertainty. Growth mindsets, the propensity to view traits and abilities as malleable rather than fixed (Dweck & Yeager, 2019), are associated with lower psychological distress (Burnette, Knouse, Vavra, O’Boyle, & Brooks, 2020; Schleider & Weisz, 2018). Furthermore, synergistic mindset interventions more effectively promote adolescent mental health than each individual component (Yeager et al., 2022).

The training’s final component targeted *repetitive negative thinking.* In both clinical (Krain et al., 2008) and nonclinical populations (Buhr & Dugas, 2002; Ladouceur, Gosselin, & Dugas, 2000), individuals with high IU are more likely to engage in repetitive negative thinking such as worry and rumination. Repetitive negative thinking both mediates and moderates the association between IU and anxiety and depression symptoms (Dar, Iqbal, & Mushtaq, 2017; Yook, Kim, Suh, & Lee, 2010). Providing strategies to prevent repetitive negative thinking should therefore potentiate the IU-focused training’s impact.

## The current study

The novel Uncertainty-Mindset Training’s effects on emerging adults’ (18–24 years, *M_age_* = 22.2, *SD_age_* = 1.3) IU and mental health, as primary preregistered outcomes (https://osf.io/fztqr), were followed-up across three months. This study compared the effects of the Uncertainty-Mindset Training (*n* = 103) to a Psychoeducation Training (*n* = 106) and No-Training (*n* = 50) control group, testing the following preregistered hypotheses: *Hypothesis 1 –* Self-reported IU at baseline would be cross-sectionally associated with greater depression and anxiety symptoms, and negative affect. *Hypothesis 2 –* The Uncertainty-Mindset Training would lead to the greatest improvements in IU and growth mindsets (H2a), and the greatest reductions in depression and anxiety symptoms, and negative affect (H2b). *Hypothesis 3 –* The effect of training group on mental health and negative affect changes would be partially accounted for by reductions in IU.

In addition to these outcomes, the study explored the impact of the Uncertainty-Mindset Training on functional impairment. Assessing functional impairment is essential to capture the training’s potential for meaningful impact, as it is indicative of future mental ill-health (Andrews & Schweizer, 2023) and can highlight difficulties faced in daily life, even by individuals with low psychological symptoms (Wille, Bettge, Wittchen, Ravens-Sieberer, & The BELLA study group, 2008). We also explored whether individuals’ initial levels of repetitive negative thinking, growth mindsets, and reappraisal tendency moderated training effects on anxiety symptoms, depression symptoms, and negative affect. While this study sought to additionally assess behavioral responses to uncertainty using a decision-under-uncertainty task, these results are reported in the supplementary materials document, given that the behavioural task used was not sensitive in the current sample and its interpretability was limited.

## Methods

### Design

The current study employed a three (Group: Uncertainty-Mindset Training, Psychoeducation Training, No-Training) by five (Time: baseline, post-assessment, 1-week, 1-month, 3-month) mixed between-within design to investigate the efficacy of the Uncertainty-Mindset Training in improving a range of primary (i.e. IU and mental health) and secondary (i.e. growth mindsets, negative affect, and functional impairment) outcomes. As per the study preregistration (https://osf.io/fztqr), participants were randomly assigned to either the Uncertainty-Mindset Training or Psychoeducation Training. Given that the Psychoeducation Training had not been previously tested to determine if it has any effects on the outcome variables above natural changes across time, a nonrandomized No-Training group was subsequently recruited as an additional comparison group, as specified in the updated preregistration (https://osf.io/m6r5y).

### Participants

170 participants were required to have 85% power to detect an effect of the Uncertainty-Mindset Training compared to the Psychoeducation Training, assuming an α of 0.05 and a small effect size (*d* = 0.18; based on a meta-analysis of mindset mental health interventions; Burnette et al., 2022). The target sample size was therefore 212 participants, conservatively estimating an attrition rate of 25% at the 3-month follow-up (Kothe & Ling, 2019). An additional 50 participants were recruited to form the no-training group in line with the updated preregistration.

At baseline, 259 participants who met the following inclusion criteria were recruited from Prolific: a) 18–24 years-old, b) able to understand and read English with native fluency, c) no history of traumatic brain injury, d) no learning difficulties, e) live in Australia, Canada, Ireland, New Zealand, South Africa, the United Kingdom, or the United States of America, f) completed a minimum of 20 prior studies on Prolific (to reduce attrition rates; Palan & Schitter, 2018), and g) did not fail more than two attention checks at any time point.

All participants were compensated at GBP9/h. For completing Phase 1 (baseline and immediate post-assessment), participants in the Uncertainty-Mindset and Psychoeducation training groups received £9 and No-Training participants received £6. Participants across all groups received £3.5 for completing each of the 1-week (Phase 2), 1-month (Phase 3), and 3-month (Phase 4) follow-ups.

### Measures

#### Intolerance of uncertainty

Trait intolerance of uncertainty was assessed with the 12-item Intolerance of Uncertainty Scale (IUS-12; Carleton, Norton, & Asmundson, 2007). Participants indicated how much each item (e.g. ‘I must get away from all uncertain situations’) resonated with them from 1 (not at all) to 5 (entirely). Total scores on the IUS-12 range from 12 to 60, with higher scores indicating greater IU, and are strongly correlated (*r* = .96) with the original 27-item version of the measure (Carleton et al., 2007). The IUS-12 also demonstrates comparable satisfactory psychometric validity to the IUS-27 (Khawaja & Yu, 2010). Acceptable internal consistency was demonstrated for the present sample (ω = .89).

#### Mental health (anxiety and depression)

The 7-item Generalised Anxiety Disorder Scale (GAD-7; Spitzer, Kroenke, Williams, & Löwe, 2006) and 8-item Patient Health Questionnaire (PHQ-8; Kroenke et al., 2009) were used to assess anxiety and depression symptoms, respectively. Participants reported the frequency with which they experience each symptom of generalized anxiety (e.g. ‘feeling nervous, anxious, or on edge’) and depression (e.g. ‘little interest or pleasure in doing things’) within the past 2 weeks. These items were rated from 0 (not at all) to 3 (nearly every day) and summed to a total of increasing symptom severity, which falls between 0 and 21 for the GAD-7, and 0 and 24 for the PHQ-8. Both scales exhibit satisfactory construct and criterion validity (Kroenke et al., 2009; Spitzer et al., 2006). Internal consistency was acceptable in the present sample for the GAD-7 (ω = .91) and PHQ-8 (ω = .88).

#### Uncertainty-related functional impairment

The functional impacts of IU were assessed with five items based on the CALIS (Lyneham et al., 2013). Participants rated, from 0 (never/very rarely) to 4 (very often/always), how often feeling uncertain makes it difficult to: interact with friends, interact with strangers, succeed in the workplace, succeed in higher education or professional/vocational training, and enjoy leisure activities/hobbies. Higher scores on this scale, ranging from 0 to 20, indicate greater uncertainty-related functional impairment. This measure showed acceptable internal consistency (ω = .75).

#### Growth uncertainty tolerance mindsets

Participants responded to one item adapted from an existing anxiety mindset measure (Schroder, Dawood, Yalch, Donnellan, & Moser, 2016): ‘Your ability to cope with uncertainty is something about you that you cannot change very much’ from 1 (Strongly Disagree [it can be changed]) to 6 (Strongly Agree [it cannot be changed]). Scores were reverse-coded such that a higher rating indicated greater growth mindsets about uncertainty tolerance.

#### Negative affect

Visual analogue scales are widely used to measure momentary mood, with distress and anxiety being core dimensions of negative affect. In the current study, participants responded on two visual analogue scales to the question: ‘How do you feel right now?’ The first scale was rated ‘-100 (Extremely happy/pleasant)’ to ‘100 (Extremely distressed/upset)’. The second scale was rated ‘-100 (Extremely relaxed)’ to ‘100 (Extremely anxious)’. The scales were combined to form a composite negative affect score, wherein higher scores indicate greater negative affect. Similar negative affect visual analogue scales have been successfully used in previous work (e.g. Abend, Dan, Maoz, Raz, & Bar-Haim, 2014; Grunewald, Deng, Wertz, & Schweizer, 2022).

#### Repetitive negative thinking

The 10-item Repetitive Thinking Questionnaire (RTQ-10) assessed worry and rumination. Participants indicated how true each item (e.g. ‘once I start thinking about the situation, I can’t stop’) is of their typical response to feeling distressed, from 1 (not at all true) to 5 (very true; McEvoy, Mahoney, & Moulds, 2010). The RTQ-10, which has previously been psychometrically validated (McEvoy et al., 2010), also demonstrated good internal consistency in the present sample (ω = .91).

#### Reappraisal tendency

The tendency to use cognitive reappraisal to alter one’s emotional experiences was assessed with the Cognitive Reappraisal subscale of the 10-item Emotion Regulation Questionnaire (ERQ), which has good convergent and discriminant validity (Gross & John, 2003). Participants responded to items (e.g. ‘I control my emotions by changing the way I think about the situation I’m in’) from 1 (strongly disagree) to 7 (strongly agree; Gross & John, 2003). Internal consistency of the ERQ reappraisal subscale in the present sample was acceptable (ω = .81).

#### Training acceptability

To measure the acceptability of the Uncertainty-Mindset and Psychoeducation trainings, participants rated the following three items from 0 (not at all) to 3 (very much): ‘The information in the text and videos was easily understandable’, ‘The information presented was useful to me’, and ‘I would recommend this module to a friend’. The items were adapted from existing questions assessing online intervention acceptability (Shapiro et al., 2023).

### Training modules

#### Uncertainty-Mindset Training

The 20–30-minute online single-session mindset training provided information in both written text and video formats. The training followed Schleider et al.’s (2020) BEST Framework for SSOIs for youth. Specifically, we: 1) utilised ***B**rain science* (e.g. explaining neuroplasticity), 2) ***E**mpowered youth as ‘helpers’* by highlighting that engaging with the training would help us improve the module to better help others, 3) included ***S**aying-is-believing questions* where participants were asked to consider how the presented information relates to their own experiences and give advice to other youth, and 4) provided ***T**estimonials* of how engaging with uncertainty has benefited real people, along with describing *evidence* from expert scientists of claims made.

#### Psychoeducation Training

The 20–30-minute information module contained content about cognitive biases, emotion regulation, the importance of social connections, forming good habits, and the benefits of exercise and sleep. Similar to the Uncertainty-Mindset Training, information was presented via written text and videos. Additionally, the same interactive questions were included, where participants were asked to provide advice to peers facing age-relevant uncertainties, assist with time-matching modules, and control for the effect of being asked to provide written responses. The question content was thematically appropriate for this training too, as it could be interpreted as applying to content on social connections, forming good habits, and biases.

### Procedure

This study was administered in four phases. Phase 1 included three components: a) the baseline assessment, b) either the Uncertainty-Mindset Training, Psychoeducation Training, or No-Training, and c) the post-assessment. Participants were randomly allocated to either the Uncertainty-Mindset or Psychoeducation group. The No-Training group was recruited one month later. Phase 1 took 1 hour for the Uncertainty-Mindset and Psychoeducation groups and 40 minutes for the No-Training group. Phases 2, 3, and 4 (20 minutes each for all three groups) were completed 1 week, 1 month, and 3 months after Phase 1, respectively.

Four attention checks were included throughout Phase 1, and three were incorporated into each follow-up phase. Only participants who answered at least two attention-check questions correctly received payment.

### Data analysis

R (Version 4.4.2) was used to conduct the following analyses. Frequentist and Bayesian statistics were reported, with the latter included to interrogate the strength of the evidence. The significance threshold for the frequentist analyses was *p* < .025 (α = .05/2), which was Bonferroni-corrected to account for the two outcome domains (IU and mental health). Bayes Factors (*BF_10_*), the ratio of evidence for the experimental hypothesis over the null hypothesis (H_1_:H_0_), were estimated using Bayesian Information Criterion approximation. Bayes factors are interpreted as anecdotal (1–3), moderate (3–10), strong (10–30), very strong (30–100), and extreme (>100) evidence for experimental hypothesis, and anecdotal (0.33–1), moderate (0.33–0.10), strong (0.10–0.03), very strong (0.03–0.01), and extreme (<0.01) evidence for the null hypothesis. Cohen’s |*d*| are interpreted as |*d*| = 0.2 (small), |*d*| = 0.5 (medium), and |*d*| ≥ 0.8 (large). As per the preregistered analysis plan, and given that the groups did not significantly differ on demographic characteristics (Table 1) or baseline scores (Supplementary Table S1), no covariates were included in the following models.

**Table 1.** Participant demographic characteristics

Variable	Total sample (*N* = 259)	Mindset training (*n* = 103)	Psychoeducation (*n* = 106)	No-training (*n* = 50)	F/χ^2^
Age (years)					1.26
M (±SD)	22.2 (±1.3)	22.0 (±1.4)	22.3 (±1.3)	22.3 (±1.4)	
Range	18–24	18–24	19–24	18–24	
Gender, n (%)					0.25
Female	171 (66.0%)	69 (67.0%)	69 (65.1%)	33 (66.0%)	
Male	86 (33.2%)	32 (31.1%)	37 (34.9%)	17 (34.0%)	
Other	1 (0.4%)	1 (1.0%)	0 (0.0%)	0 (0.0%)	
Prefer not to say	1 (0.4%)	1 (1.0%)	0 (0.0%)	0 (0.0%)	
Ethnicity, n (%)					2.57
Aboriginal or Torres Strait Islander	1 (0.4%)	1 (1.0%)	0 (0.0%)	0 (0.0%)	
Asian	13 (5.0%)	6 (5.8%)	3 (2.8%)	4 (8.0%)	
Black	209 (80.7%)	81 (78.6%)	87 (82.1%)	41 (82.0%)	
Mixed	7 (2.7%)	3 (2.9%)	3 (2.8%)	1 (2.0%)	
White	26 (10.0%)	10 (9.7%)	1 (0.9%)	4 (8.0%)	
Prefer not to say	3 (1.2%)	2 (1.9%)	12 (11.3%)	0 (0.0%)	
Highest Education Level, n (%)					4.61
Primary School	1 (0.4%)	1 (1.0%)	0 (0.0%)	0 (0.0%)	
High School	88 (34.0%)	29 (28.2%)	44 (41.5%)	15 (30.0%)	
Professional/Vocational Training	18 (6.9%)	7 (6.8%)	8 (7.5%)	3 (6.0%)	
University	150 (57.9%)	64 (62.1%)	54 (50.9%)	32 (64.0%)	
Prefer not to say	2 (0.8%)	2 (1.9%)	0 (0.0%)	0 (0.0%)	
Mental Health Diagnosis, n (%)					1.61
Yes	28 (10.8%)	8 (7.8%)	14 (13.2%)	6 (12.0%)	
No	226 (87.3%)	92 (89.3%)	90 (84.9%)	44 (88.0%)	
Prefer not to say	5 (1.9%)	3 (2.9%)	2 (1.9%)	0 (0.0%)	

*Note:* No group differences in demographic characteristics were observed, as no *F* or *χ^2^* statistics were significant.

Generalized linear models were used to investigate *Hypothesis 1.* These models included baseline IU as the fixed factor, and mental health (anxiety symptoms, depression symptoms, and negative affect) as outcomes. *Hypothesis 2* was investigated with mixed models using restricted maximum likelihood estimation. These included Group and Time as fixed effects that were allowed to interact, ID as a random factor, and IU, growth mindsets, anxiety, depression, and negative affect as outcomes. Significant Group × Time interactions were interrogated by investigating the effect of Time on the outcome of interest within each group separately. Where more than one group showed significant Time effects, these two groups were directly compared in a follow-up mixed model analysis including these groups only. To examine *Hypothesis 3*, we ran mediation models including change in IU for each follow-up period as the mediator, Group as the independent variable, and change in mental well-being outcomes as the dependent variables.

As per the preregistered exploratory analyses, models testing hypotheses 1 and 2 were run including functional impairment as the outcome. Finally, the exploratory analyses aimed at examining the potential moderating effects of baseline growth mindsets, repetitive negative thinking, and reappraisal tendency on the associations between group and mental health changes across time were explored with generalized linear models.

## Results

### Participant characteristics

Following the exclusion of three participants who reported learning difficulties, and five participants who used AI to respond to the short answer questions in the trainings, the final sample was comprised of 259 participants at baseline. Retention was good across the post-assessment (257; 99.2%) and follow-ups: 251 (96.9%) at 1-week, 227 (87.6%) at 1-month, and 219 (84.6%) at 3-months (Supplementary Figure S1). The majority of the sample were Black (80.7%), female (66.0%), had completed university (57.9%), and did not have a current or past mental health diagnosis (87.3%; Table 1). The groups did not significantly differ on demographic and clinical characteristics (Table 1) or on baseline scores (Supplementary Table S1).

### Baseline associations

**Figure 1.**
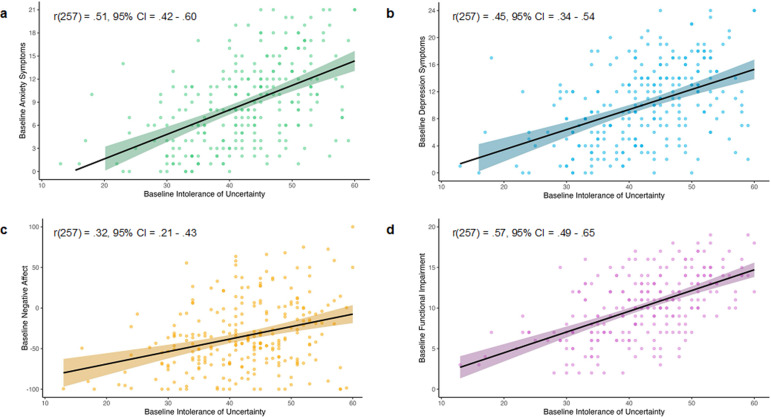
Relationships of intolerance of uncertainty with mental health symptoms, negative affect, and functional impairment. *Note:* The figure illustrates the association between intolerance of uncertainty (IUS-12) and (a) *symptoms of anxiety* (GAD-7), (b) *symptoms of depression* (PHQ-8), (c) *negative affect* (two visual analogue scales), and (d) *functional impairment* due to uncertainty (modified CALIS). Darker plot points indicate multiple participants with the same set of scores. The shaded area around the trendline represents the 95% confidence interval.

At baseline, IU was significantly associated with more symptoms of anxiety (*F*(1, 257) = 91.78, *p* < .001, *R* ^2^ = .26, *BF_10_* > 100; Figure 1a) and depression (*F*(1, 257) = 64.34, *p* < .001, *R* ^2^ = .20, *BF_10_* > 100; Figure 1b), higher negative affect (*F*(1, 257) = 29.29, *p* < .001, *R* ^2^ = .10, *BF_10_* > 100; Figure 1c), and functional impairment in response to uncertainty (*F*(1, 257) = 126.80, *p* < .001, *R* ^2^ = .33, *BF_10_* > 100; Figure 1d).

### Training effects

#### Intolerance of uncertainty

**Figure 2.**
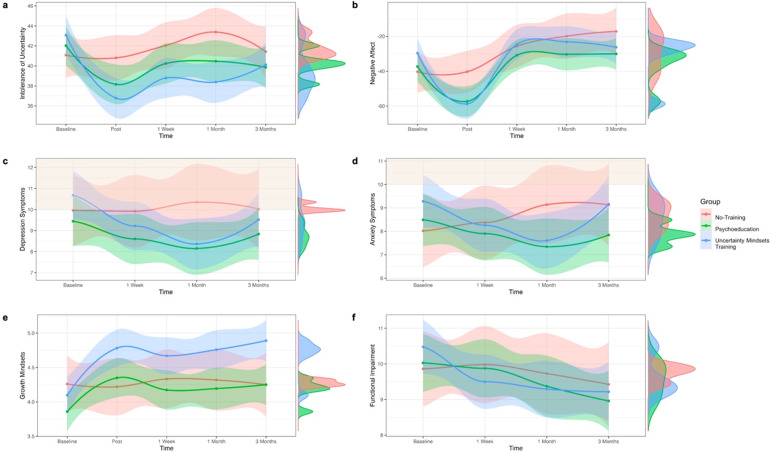
Training effects on intolerance of uncertainty, growth mindsets, mental health symptoms, negative affect, and functional impairment. *Note:* The figure shows graphs with means (dot points), 95% confidence intervals (shaded area), and distributions (density graphs on the side) for each outcome measure across the three groups (i.e. No-Training, Psychoeducation, and Uncertainty-Mindset Training) over time (i.e. baseline, post-assessment, 1-week, 1-month, and 3-months) for the following outcomes of interest: (a) *intolerance of uncertainty* (IUS-12); (b) *negative affect* (two visual analogue scales); (c) *symptoms of depression* (PHQ-8; clinical cut-off range indicated in orange); (d) *symptoms of anxiety* (GAD-7; clinical cut-off range indicated in orange); (e) *growth mindsets* about uncertainty tolerance (single item); and (f) *functional impairment* due to uncertainty (modified CALIS).

Mixed-effects models showed that the Uncertainty-Mindset Training significantly reduced IU up to 1 month later, with significant Group × Time interactions at the post-training, 1-week, and 1-month follow-ups (Table 2, Figure 2a). Breaking down these interactions showed small-medium effects of Time in the Psychoeducation group at post-training and 1-week, and large effects in the Uncertainty-Mindset Training group at post-training, 1-week, and 1-month (Table 3). While there was a medium effect of Time in the No-Training group at 1-month, this was in the direction of increased IU. Directly comparing the two training groups showed a greater decrease in IU in the Uncertainty-Mindset Training compared to the Psychoeducation Training at each of these time points (post-training: *F*(1,206) = 5.87, *p* = .016, *BF_10_ =* 2.28; 1-week: *F*(1, 203) = 6.41, *p* = .012, *BF_10_ =* 3.04; 1-month: *F*(1,191) = 6.13, *p* = .014, *BF_10_ =* 3.14).

**Table 2.** Mixed effects models investigating the effects of group and time on intolerance of uncertainty, growth mindsets, mental health symptoms, negative affect, and functional impairment at the post-assessment, 1-week, 1-month, and 3-month follow-ups

	Post-assessment				1-Week				1-Month				3-Months			
	*F*	*df*	*p*	*BF_10_*	*F*	*df*	*p*	*BF_10_*	*F*	*df*	*p*	*BF_10_*	*F*	*df*	*p*	BF_10_
Intolerance of uncertainty																
Group	0.22	(2,256)	.799	0.027	0.11	(2,256)	.894	0.022	0.47	(2,257)	.623	0.028	0.06	(2,260)	.944	0.022
Time	57.45	(1,255)	<.001	>100	15.08	(1,251)	<.001	>100	6.98	(1,236)	.009	>100	7.80	(1,232)	.006	70.27
Group × Time	12.72	(2,255)	<.001	>100	10.11	(2,251)	<.001	>100	12.38	(2,236)	<.001	>100	3.46	(2,232)	.033	0.670
Growth mindsets																
Group	1.66	(2,256)	.193	0.002	2.51	(2,257)	.083	0.005	2.43	(2,254)	.090	0.004	3.72	(2,259)	.026	0.011
Time	26.66	(1,255)	<.001	>100	12.00	(1,253)	<.001	58.41	13.34	(1,235)	<.001	>100	13.82	(1,239)	<.001	>100
Group × Time	7.19	(2,255)	<.001	0.377^a^	2.32	(2,253)	.101	0.006	3.12	(2,235)	.046	0.012	4.59	(2,238)	.011	0.069^a^
Anxiety symptoms																
Group	–	–	–	–	0.41	(2,258)	.662	0.011	0.30	(2,258)	.741	0.010	0.67	(2,258)	.515	0.015
Time	–	–	–	–	2.30	(1,252)	.130	0.325	1.94	(1,237)	.165	0.999	0.07	(1,235)	.795	0.048
Group × Time	–	–	–	–	1.52	(2,252)	.220	0.024	5.46	(2,237)	.005	1.70	1.83	(2,234)	.163	0.064
Depression symptoms																
Group	–	–	–	–	0.94	(2,257)	.393	0.020	1.19	(2,259)	.307	0.024	0.90	(2,258)	.407	0.020
Time	–	–	–	–	7.45	(1,252)	.007	9.85	6.40	(1,239)	.012	22.08	1.41	(1,235)	.236	0.209
Group × Time	–	–	–	–	1.52	(2,252)	.221	0.025	4.43	(2,239)	.013	0.744^a^	1.33	(2,234)	.267	0.044
Negative affect																
Group	0.66	(2,256)	.518	0.663	1.00	(2,257)	.369	1.07^a^	1.00	(2,260)	.371	1.17^a^	0.70	(2,257)	.498	0.857
Time	60.28	(1,255)	<.001	>100	7.79	(1,253)	.006	8.34	11.62	(1,245)	<.001	27.47	11.88	(1,240)	<.001	18.18
Group × Time	14.08	(2,255)	<.001	>100	0.70	(2,253)	.500	1.24^a^	1.60	(2,245)	.205	3.53^a^	2.74	(2,238)	.067	12.28^a^
Functional impairment																
Group	–	–	–	–	0.02	(2,257)	.985	0.003	0.12	(2,257)	.888	0.004	0.15	(2,256)	.864	0.004
Time	–	–	–	–	2.13	(1,252)	.146	0.168	7.48	(1,237)	.007	7.37	11.38	(1,234)	<.001	58.42
Group × Time	–	–	–	–	1.76	(2,252)	.173	0.021	1.29	(2,237)	.278	0.013	0.65	(2,233)	.523	0.010

*Note:* Group = No-Training versus Psychoeducation versus Uncertainty-Mindset Training. Time = baseline, post-assessment, 1-week, 1-month, and 3-months. Intolerance of uncertainty = IUS-12. Growth mindsets = single item. Anxiety symptoms = GAD-7. Depression symptoms = PHQ-8. Negative affect = two visual analogue scales. Functional impairment due to uncertainty = modified CALIS. The significance threshold utilised for the frequentist analyses is *p* < .025. Bayes factors (*BF_10_*) are interpreted as anecdotal (1–3), moderate (3–10), strong (10–30), very strong (30–100), and extreme (>100) evidence for the experimental hypothesis, and anecdotal (0.33–1), moderate (0.33–0.10), strong (0.10–0.03), very strong (0.03–0.01), and extreme (<0.01) evidence for the null hypothesis.

a Indicates where there is a discrepancy between the outcomes of the frequentist and Bayesian analyses.

**Table 3.** Time effects on intolerance of uncertainty and negative affect within each group at each follow-up assessment

	Post-assessment					1-Week					1-Month					3-Months				
	*β*	*CI*	*p*	*d*	*BF_10_*	*β*	*CI*	*p*	*d*	*BF_10_*	*β*	*CI*	*p*	*d*	*BF_10_*	*β*	*CI*	*p*	*d*	*BF_10_*
Intolerance of uncertainty																				
Mindset training	−0.59	[−0.75, −0.44]	<.001	1.28	>100	−0.46	[−0.62, −0.30]	<.001	0.91	>100	−0.47	[−0.64, −0.30]	<.001	0.86	>100	−0.36	[−0.54, −0.17]	<.001	0.60	>100
Psychoeducation	−0.38	[−0.49, −0.26]	<.001	0.79	>100	−0.19	[−0.32, −0.05]	.007	0.38	4.85	−0.17	[−0.34, −0.01]	.038	0.32	1.32^a^	−0.21	[0.38, −0.03]	.022	0.36	2.25
No-training	−0.03	[−0.22, 0.15]	.716	0.06	0.204	0.12	[−0.07, 0.30]	.216	−0.18	0.395	0.29	[0.09, 0.50]	.008	−0.41	8.08	0.09	[−0.17, 0.35]	.517	−0.11	0.311
Negative affect																				
Mindset training	−0.71	[−0.88, −0.54]	<.001	1.29	>100	0.12	[−0.09, 0.33]	.255	−0.16	1.58^a^	0.15	[−0.08, 0.37]	.199	−0.19	2.10^a^	0.09	[−0.13, 0.30]	.445	−0.11	1.23^a^
Psychoeducation	−0.49	[−0.64, −0.34]	<.001	0.88	>100	0.14	[−0.07, 0.35]	.190	−0.19	2.02^a^	0.14	[−0.07, 0.36]	.192	−0.20	2.14^a^	0.16	[−0.07, 0.39]	.168	−0.22	2.49^a^
No-training	0.00	[−0.15, 0.15]	.995	−0.00	0.736	0.36	[0.06, 0.67]	.020	−0.44	24.73	0.47	[0.20, 0.74]	.001	−0.61	>100	0.58	[0.26, 0.90]	<.001	−0.72	>100

*Note:* Intolerance of uncertainty = IUS-12. Negative affect = two visual analogue scales. The significance threshold utilised for the frequentist analyses is *p* < .025. Bayes factors (*BF_10_*) are interpreted as anecdotal (1–3), moderate (3–10), strong (10–30), very strong (30–100), and extreme (>100) evidence for the experimental hypothesis, and anecdotal (0.33–1), moderate (0.33–0.10), strong (0.10–0.03), very strong (0.03–0.01), and extreme (<0.01) evidence for the null hypothesis.

a Indicates where there is a discrepancy between the outcomes of the frequentist and Bayesian analyses.

#### Mental health

Comparing the effect of training group on symptoms of depression and anxiety at the 1-month follow-up revealed significant Group × Time interactions (Table 2, Figure 2b, c). However, it should be noted that Bayesian analyses only supported the interaction for anxiety symptoms (Table 2). To break down these interactions, the effect of Time was investigated in each group separately, revealing that the interaction was due to medium size reductions in symptoms of anxiety (*β* = −0.31, CI [−0.48, −0.15], *p* < .001, *d* = .50, *BF_10_* = 52.05) and depression (*β* = −0.39, CI [−0.57, −0.21], *p* < .001, *d* = .60, *BF_10_* > 100) in the Uncertainty-Mindset group, which were not observed in the other two groups (*p*s > .025; Supplementary Table S2). However, these benefits were not maintained at 3-months (Table 2).

Importantly, the Uncertainty-Mindset Training did appear to improve mental health by reducing IU, as evidenced by a significant indirect effect of change in IU on mental health improvements at 1-month (Supplementary Table S3). This mediating effect persisted at 3-months for anxiety symptoms (Supplementary Table S3).

#### Negative affect

Like mental health, negative affect varied across training groups over time as shown by significant Group × Time interactions (Table 2; Figure 2d). It should be noted that while the effect was observed for frequentist and Bayesian analyses immediately post-training, only Bayesian statistics supported the effects at the 1-week, 1-month, and 3-month follow-ups. To unpack these significant interactions, the effect of Time was investigated in each group separately. Immediately post-training, both the Uncertainty-Mindset and Psychoeducation groups showed large effects of Time on negative affect, with self-reported distressed and anxious mood being lower following completion of the training modules (Table 3). The Bayes factor suggests that this reduction in negative affect was greater in the Uncertainty-Mindset group compared to the Psychoeducation group (*F*(1,206) = 3.88, *p* = .050, *BF_10_ =* 4.01).

Negative affect increased at all subsequent time points for all groups (Table 3). However, when comparing groups directly at the pairwise level, evidence emerged for the superiority of the Uncertainty-Mindset Training in protecting mood compared to No-Training at the 1-week (*F*(1,149) = 1.39, *p* = .240, *BF_10_ =* 2.30), 1-month (*F*(1,143) = 2.89, *p* = .092, *BF_10_ =* 5.25), and 3-month (*F*(1,134) = 6.22, *p* = .014, *BF_10_ =* 27.43) follow-ups, and compared to the Psychoeducation Training at the 3-month follow-up (*F*(1,194) = 0.26, *p* = .608, *BF_10_ =* 1.07). Psychoeducation also protected mood compared to No-Training at 1-week (*F*(1,153) = 0.99, *p* = .320, *BF_10_ =* 1.95), 1-month (*F*(1,147) = 2.76, *p* = .098, *BF_10_ =* 4.96), and 3-months (*F*(1,148) = 3.67, *p* = .069, *BF_10_ =* 7.38).

Again, these group differences in negative affect changes were partially accounted for by changes in IU (Supplementary Table S3).

#### Functional impairment

There was a significant reduction in functional impairment from baseline to the 1-month and 3-month follow-ups, but this effect did not differ across the groups (Table 2; Figure 2f).

#### Growth mindsets

Group-related differences in growth mindsets emerged immediately post-training and at the 3-month follow-up, as evidenced by significant (frequentist but not Bayesian analyses) Group × Time interactions (Table 2; Figure 2e), with both training groups showing significant increases in growth mindsets across time (Supplementary Table S4).

### Moderating effects

Baseline growth mindsets, repetitive negative thinking, and reappraisal tendency did not moderate training effects on anxiety symptoms, depression symptoms, or negative affect at any follow-up assessment (*p* > .025).

### Training acceptability

The Uncertainty-Mindset and Psychoeducation trainings were rated as highly acceptable, with most indicating that they very much (i.e. 3) agreed with each facet of acceptability. Specifically, participants agreed that the trainings were easily understandable (Uncertainty-Mindset: *M* = 2.96, *SD* = 0.20; Psychoeducation: *M* = 2.88, *SD* = 0.43), presented useful information (Uncertainty-Mindset: *M* = 2.86, *SD* = 0.42; Psychoeducation: *M* = 2.90, *SD* = 0.39), and they would recommend them to a friend (Uncertainty-Mindset: *M* = 2.86, *SD* = 0.45; Psychoeducation: *M* = 2.90, *SD* = 0.36).

## Discussion

IU is a transdiagnostic risk factor for youth mental health problems (Morriss, 2025). Improving individuals’ capacity to tolerate uncertainty is a promising target for prevention in the context of the high geopolitical, climate, and social uncertainty currently facing young people (The Lancet, 2022). To address this need, we developed a novel, easy-to-disseminate, single-session online training targeting IU in older adolescents.

This study showed that intolerance of uncertainty was associated with poorer mental health (i.e. greater anxiety and depression symptoms), greater negative affect, and worse functional impairment in emerging adults. The Uncertainty-Mindset Training substantially reduced IU (up to 1 month later) and symptoms of mental ill-health (at 1-month), increased growth mindsets (at the post-assessment and 3-months) and had a protective effect on mood (up to 3 months later). Importantly, the mental health and mood improvements were accounted for by reductions in IU. We also found that the Uncertainty-Mindset Training did not result in greater decreases in functional impairment at any follow-up, nor did training effects on mental health and negative affect depend on initial levels of repetitive negative thinking, growth mindsets, or reappraisal tendency.

In line with IU as a transdiagnostic risk factor (Mahoney & McEvoy, 2012), IU was cross-sectionally associated with greater negative affect, anxiety symptoms, and depression symptoms. While this relationship is well established in adults (Dar et al., 2017; Gentes & Ruscio, 2011) and in children and younger adolescents (Osmanağaoğlu, Creswell, & Dodd, 2018; Ye et al., 2023), our results add to the growing literature on emerging adulthood, a time of especially significant uncertainty (Arnett et al., 2014; Blakemore, 2019). Interestingly, we also found that IU was associated with greater uncertainty-related functional impairment across domains. This is an important finding, as the functional impacts of high IU are poorly understood (Shapiro, Gros, & McCabe, 2020). Collectively, these results support IU as a useful prevention and intervention target.

While previous SSOI research has pointed to IU potentially not being amenable to rapid change (Shapiro et al., 2023), our study indicates that it can be reduced quickly. Indeed, our ultra-brief self-guided training led to substantial reductions in IU immediately post-training (*d* = 1.28) and at 1-week (*d* = 0.91) that were maintained across a 1-month period (*d* = 0.86). Our training’s success may be attributed to explicitly highlighting the potential future rewards of engaging with uncertainty, especially in the context of rising negative reactivity to uncertainty (Carleton, Desgagné, Krakauer, & Hong, 2019) and the lack of popular awareness of its associated benefits (Alquist & Baumeister, 2024; Wilson, Centerbar, Kermer, & Gilbert, 2005). Heightened reward salience fosters approach behavior (Renz, Pillny, & Lincoln, 2022) and thus may encourage engagement with uncertainty which, in turn, can improve uncertainty attitudes (Alquist & Baumeister, 2024). However, the training’s superiority in reducing IU relative to the control groups did not persist at 3-months post-training. While research on whether these brief online universal trainings can have lasting effects on IU is limited, future studies should investigate how the Uncertainty-Mindset Training can be improved to extend its benefits.

The Uncertainty-Mindset Training exhibited promising clinical utility given that it reduced anxiety (*d* = 0.50) and depression (*d* = 0.60) symptoms 1 month post-training and had a protective effect on mood up to 3 months post-training. While the Uncertainty-Mindset Training group was the only group to experience a significant reduction in mental ill-health, their reported symptom levels remained higher than those of the Psychoeducation group at each assessment. It is therefore possible that the Uncertainty-Mindset Training’s mental health improvements could simply be attributed to regression-to-the-mean. However, this explanation is countered by building on the science for behavioral change framework that requires intervention development to demonstrate that changes in the clinical outcome of interest are associated with changes in the intervention’s proposed mechanism of action (Sumner et al., 2018). This was demonstrated in the current study with the observed mediating effect of changes in the training’s proposed mechanism of action (i.e. IU) on changes in mental health and negative affect.

There was a significant increase in growth mindsets for the Uncertainty-Mindset group immediately post-training (*d* = −0.87), in line with other SSOIs (Schleider & Weisz, 2016). While the assessment of growth mindsets at longer-term follow-ups is limited, a notable exception is a study that showed increases in intelligence growth mindsets emerging at 3-months, and emotion and behavior growth mindsets at 6-months (Verberg, Helmond, Otten, & Overbeek, 2022). Here, our Uncertainty-Mindset group showed an effect of training again at 3-months (*d* = −0.77), whereas the effect became small in the Psychoeducation group. This suggests that adopting growth mindsets may also require real-world experiences of malleability beyond the training.

Another meaningful indicator of the potential for clinical impact is functioning. The Uncertainty-Mindset Training did not reduce functional impairment greater than either control group at any follow-up. While functional outcomes are often ignored when evaluating SSOIs, this result is consistent with the broader intervention literature, which shows that improvements in functioning often trail symptom improvements (Andrews & Schweizer, 2023). However, the brief and non-personalised nature of single-session online trainings may limit their impact on functional impairment (Depp, Perivoliotis, Holden, Dorr, & Granholm, 2019).

### Clinical implications and future directions

The Uncertainty-Mindset Training demonstrated promising clinical utility in its ability to reduce symptoms of mental ill-health by reducing IU in emerging adults. Given the financial and temporal barriers that often prevent youth from accessing mental health care (Brown et al., 2016), its potential for impact is substantial as a brief training that can be delivered at scale at no cost to the users. It must be noted that while these trainings are not intended to replace traditional therapeutic interventions given the modest reductions in symptoms and likely limited utility for individuals with more severe symptoms, they may be especially useful if applied at the population level to protect and improve youth mental health. Being able to reliably change population mental health symptom means, even if by a small amount, would have important benefits for public health more broadly (Cardamone-Breen et al., 2018; Rose, 1993). In addition to the advantages of a universal approach, tailoring training content to specific types of uncertainty (e.g. in healthcare or relationship contexts) and targeting distribution towards specific populations (e.g. individuals with chronic illnesses or younger adolescents) may extend the training’s effects. It is however important to recognize that while the Uncertainty-Mindset Training can foster mental well-being on an individual level, addressing larger societal problems (e.g. cost-of-living and housing crises, systemic oppression, ethnoracial marginalization, and forced migration) is essential to halt the rapid rise in youth mental health problems.

### Strengths and limitations

Importantly, the study pre-registered predictions and included a diverse sample, with a majority of participants (87%) residing outside of high-income countries. On the other hand, the measure of functional impairment was adapted from its original version, though preliminary evaluation indicated that it had acceptable internal consistency. Additionally, uncertainty tolerance growth mindsets were only assessed with a single item, which may not be sufficiently sensitive to individual variance or changes across time. Another limitation is that while participants were randomised to the Uncertainty-Mindset Training and Psychoeducation groups, they were not randomly allocated to the No-Training group. Mitigating the non-random allocation of No-Training participants is that they were also recruited from Prolific and showed no significant differences in demographic or clinical characteristics compared with the other groups. Conducting a randomised control trial is an important next step before large-scale dissemination. Additionally, given the inclusion of interactive questions in the Psychoeducation Training asking participants to provide advice on facing uncertainty, it is possible that the conclusions regarding the relative effects of the Uncertainty-Mindset Training are conservative.

## Conclusions

This proof-of-concept study demonstrated the promise of an ultra-brief single-session online training to deliver meaningful change in a transdiagnostic risk factor for mental health problems: IU. Given the rising rates of mental health problems and barriers that prevent mental health care utilisation in young people, there is a pressing need for easy-to-disseminate tools to improve youth well-being. The Uncertainty-Mindset Training addresses these needs, demonstrating promising effects for youth mental health. Replicating these findings in a definitive randomised control trial is critical.

## Supporting information

Daniels et al. supplementary material Daniels et al. supplementary material

## Supplementary material

The supplementary material for this article can be found a http://doi.org/10.1017/S0033291725102419.
