# Supplementary material for: A single session online training reduces intolerance of uncertainty and improves mental health in emerging adults
Source: Psychol Med. 2025 Dec 15;55:e377. doi: 10.1017/S0033291725102419 (PMC13058662; doi:10.1017/S0033291725102419)
Supplement: Daniels et al. supplementary material [file S0033291725102419sup001.docx]

**Supplementary Table 1: Descriptive statistics for each group across time**

| Variable | Total Sample  (*N* = 259) | Mindset Training  (*n* = 103) | Psychoeducation  (*n* = 106) | No-Training  (*n* = 50) | *F* |
| --- | --- | --- | --- | --- | --- |
| **Intolerance of uncertainty** M (±SD) |  |  |  |  |  |
| Baseline | 42.26 (±8.86) | 43.07 (±9.14) | 42.03 (±9.45) | 41.08 (±6.74) | 0.91 |
| Post-Assessment | 38.13 (±10.67) | 36.78 (±11.13) | 38.16 (±10.72) | 40.80 (±9.19) |  |
| One-Week | 40.01 (±9.99) | 38.79 (±10.06) | 40.24 (±10.46) | 42.04 (±8.52) |  |
| One-Month | 40.20 (±10.27) | 38.38 (±10.29) | 40.47 (±10.87) | 43.39 (±8.08) |  |
| Three-Months | 40.24 (±9.89) | 40.12 (±10.06) | 39.85 (±10.52) | 41.42 (±7.94) |  |
| **Anxiety Symptoms** M (±SD) |  |  |  |  |  |
| Baseline | 8.71 (±5.49) | 9.28 (±5.47) | 8.49 (±5.79) | 8.02 (±4.85) | 1.04 |
| One-Week | 8.14 (±5.75) | 8.26 (±5.94) | 7.90 (±5.82) | 8.38 (±5.25) |  |
| One-Month | 7.79 (±5.91) | 7.60 (±5.78) | 7.34 (±5.90) | 9.14 (±6.13) |  |
| Three-Months | 8.57 (±6.04) | 9.16 (±6.04) | 7.85 (±6.05) | 9.13 (±5.99) |  |
| **Depression Symptoms** M (±SD) |  |  |  |  |  |
| Baseline | 10.03 (±5.88) | 10.68 (±5.62) | 9.44 (±6.16) | 9.96 (±5.77) | 1.16 |
| One-Week | 9.10 (±5.92) | 9.22 (±5.78) | 8.60 (±6.13) | 9.92 (±5.78) |  |
| One-Month | 8.65 (±6.31) | 8.36 (±6.15) | 8.15 (±6.27) | 10.35 (±6.58) |  |
| Three-Months | 9.31 (±6.06) | 9.52 (±6.00) | 8.84 (±6.12) | 10.03 (±6.09) |  |
| **Negative Affect** M (±SD) |  |  |  |  |  |
| Baseline | -34.84 (±42.64) | -29.63 (±42.71) | -37.32 (±44.00) | -40.30 (±39.09) | 1.36 |
| Post-Assessment | -54.57 (±36.39) | -58.74 (±34.03) | -57.29 (±34.78) | -40.28 (±41.30) |  |
| One-Week | -27.35 (±46.47) | -24.59 (±46.73) | -30.83 (±48.86) | -25.61 (±40.74) |  |
| One-Month | -25.48 (±48.36) | -23.14 (±47.16) | -30.31 (±49.44) | -19.87 (±48.62) |  |
| Three-Months | -26.31 (±48.40) | -26.23 (±47.61) | -30.06 (±50.09) | -17.15 (±45.72) |  |
| **Growth Mindsets** M (±SD) |  |  |  |  |  |
| Baseline | 4.03 (±1.40) | 4.10 (±1.36) | 3.86 (±1.43) | 4.26 (±1.40) | 1.60 |
| Post-Assessment | 4.49 (±1.51) | 4.78 (±1.49) | 4.35 (±1.49) | 4.22 (±1.50) |  |
| One-Week | 4.40 (±1.48) | 4.67 (±1.33) | 4.17 (±1.54) | 4.33 (±1.58) |  |
| One-Month | 4.44 (±1.44) | 4.76 (±1.39) | 4.19 (±1.46) | 4.32 (±1.38) |  |
| Three-Months | 4.49 (±1.48) | 4.89 (±1.28) | 4.25 (±1.50) | 4.25 (±1.64) |  |
| **Functional Impairment** M (±SD) |  |  |  |  |  |
| Baseline | 10.17 (±3.94) | 10.48 (±3.91) | 10.03 (±4.12) | 9.86 (±3.65) | 0.53 |
| One-Week | 9.75 (±3.95) | 9.50 (±3.77) | 9.87 (±4.12) | 9.98 (±4.01) |  |
| One-Month | 9.41 (±4.15) | 9.29 (±4.04) | 9.37 (±4.40) | 9.73 (±3.90) |  |
| Three-Months | 9.14 (±3.98) | 9.22 (±3.78) | 8.96 (±4.32) | 9.43 (±3.61) |  |

No group differences at baseline were observed as no *F* statistics were significant.

**Supplementary Table 2: Time effects on mental health symptoms within each group at one-month**

|  | One-Month | | | | |
| --- | --- | --- | --- | --- | --- |
|  | *β* | CI | *p* | *d* | *BF_10_* |
| **Anxiety Symptoms** |  |  |  |  |  |
| Mindset Training | -0.31 | [-0.48, -0.15] | < .001 | 0.50 | 52.05 |
| Psychoeducation | -0.17 | [-0.35, 0.02] | .083 | 0.27 | 0.453 |
| No-Training | 0.23 | [-0.04, 0.50] | .099 | -0.35 | 0.780 |
| **Depression Symptoms** |  |  |  |  |  |
| Mindset Training | -0.39 | [-0.57, -0.21] | < .001 | 0.60 | > 100 |
| Psychoeducation | -0.19 | [-0.39, 0.00] | .057 | 0.30 | 0.695 |
| No-Training | 0.11 | [-0.11, 0.33] | .323 | -0.16 | 0.292 |

Time effects for anxiety and depression symptoms within each individual group (Uncertainty-Mindset Training, Psychoeducation, and No-Training). Anxiety symptoms were measured with the 7-item Generalized Anxiety Disorder Scale (Spitzer et al., 2006). Depression symptoms were measured with the 8-item Patient Health Questionnaire (Kroenke et al., 2009). The significance threshold utilised for the frequentist analyses is *p* < .025. *BF_10_* is the Bayes Factor ratio of the evidence for the alternative hypothesis over the null hypothesis (H_1_:H_0_). Bayes factors are interpreted as anecdotal (1-3), moderate (3-10), strong (10-30), very strong (30-100) and extreme (>100) evidence for H_1_, and anecdotal (*BF*_10_ = 0.33-1), moderate (0.33-0.10), strong (0.10-0.03), very strong (0.03-0.01), and extreme (<0.01) evidence for H_0_ (Andraszewicz et al., 2014). Cohen’s *d* are interpreted as |*d|* = 0.2 (small), |*d|* = 0.5 (medium), and |*d|* ≥ 0.8 (large).

**Supplementary Table 3: Mediating effect of change in intolerance of uncertainty on group differences in the change in mental health symptoms and negative affect at post-assessment, one-week, one-month, and three-months**

|  | Anxiety Symptoms | | | | Depression Symptoms | | | | Negative Affect | | | |
| --- | --- | --- | --- | --- | --- | --- | --- | --- | --- | --- | --- | --- |
|  | *b** | *SE* | *CI* | *p* | *b** | *SE* | *CI* | *p* | *b** | *SE* | *CI* | *p* |
| Group 🡪 IU change from baseline to post-assessment 🡪 Affect change from baseline to post-assessment |  |  |  |  |  |  |  |  | -0.099 | 0.032 | [-0.161, -0.038] | **.002** |
| Group 🡪 IU change from baseline to one-week 🡪 Symptom/affect change from baseline to one-week | -0.079 | 0.036 | [-0.149, -0.010] | .025 | -0.048 | 0.029 | [-0.104, 0.008] | .096 | -0.064 | 0.030 | [-0.122, -0.006] | .031 |
| Group 🡪 IU change from baseline to one-month 🡪 Symptom/affect change from baseline to one-month | -0.140 | 0.046 | [-0.230, -0.050] | **.002** | -0.098 | 0.040 | [-0.177, -0.019] | **.015** | -0.101 | 0.038 | [-0.176, -0.027] | **.008** |
| Group 🡪 IU change from baseline to three-months 🡪 Symptom/affect change from baseline to three-months | -0.093 | 0.041 | [-0.173, -0.013] | **.023** | -0.083 | 0.038 | [-0.156, -0.009] | .028 | -0.077 | 0.031 | [-0.138, -0.016] | **.014** |

*p* < .025 are bolded to indicate significant indirect effects. Intolerance of uncertainty was measured with the 12-item Intolerance of Uncertainty Scale (Carleton et al., 2007). Anxiety symptoms were measured with the 7-item Generalized Anxiety Disorder Scale (Spitzer et al., 2006). Depression symptoms were measured with the 8-item Patient Health Questionnaire (Kroenke et al., 2009). Negative affect was measured using two visual analogue scales where higher scores indicate greater negative affect (i.e., more distressed and anxious mood).

**Supplementary Table 4: Time effects on growth mindsets within each group immediately post-training and at three-months**

|  | Post-Assessment | | | | | Three-Months | | | | |  |
| --- | --- | --- | --- | --- | --- | --- | --- | --- | --- | --- | --- |
|  | *β* | CI | *p* | *d* | *BF_10_* | *β* | CI | *p* | *d* | *BF_10_* | |
| **Growth Mindsets** |  |  |  |  |  |  |  |  |  |  | |
| Mindset Training | 0.47 | [0.30, 0.63] | < .001 | -0.87 | > 100 | 0.59 | [0.37, 0.81] | < .001 | -0.77 | > 100 | |
| Psychoeducation | 0.33 | [0.19, 0.47] | < .001 | -0.63 | > 100 | 0.25 | [0.04, 0.45] | .020 | -0.34 | 0.429* | |
| No-Training | 0.03 | [-0.19, 0.14] | .743 | 0.05 | 0.032 | 0.00 | [-0.32, 0.33] | .983 | -0.00 | 0.065 | |

Time effects for growth mindsets within each individual group (Uncertainty-Mindset Training, Psychoeducation, and No-Training). Growth mindsets about uncertainty tolerance were assessed with our novel item. The significance threshold utilised for the frequentist analyses is *p* < .025. *BF_10_* is the Bayes Factor ratio of the evidence for the alternative hypothesis over the null hypothesis (H_1_:H_0_). Bayes factors are interpreted as anecdotal (1-3), moderate (3-10), strong (10-30), very strong (30-100) and extreme (>100) evidence for H_1_, and anecdotal (*BF*_10_ = 0.33-1), moderate (0.33-0.10), strong (0.10-0.03), very strong (0.03-0.01), and extreme (<0.01) evidence for H_0_ (Andraszewicz et al., 2014). * indicates where there is a discrepancy between the outcomes of the frequentist and Bayesian analyses. Cohen’s *d* are interpreted as |*d|* = -0.2 (small),

|*d|* = -0.5 (medium), and |*d|* ≥ -0.8 (large).

**Supplementary Figure 1: Participant Flow Diagram**


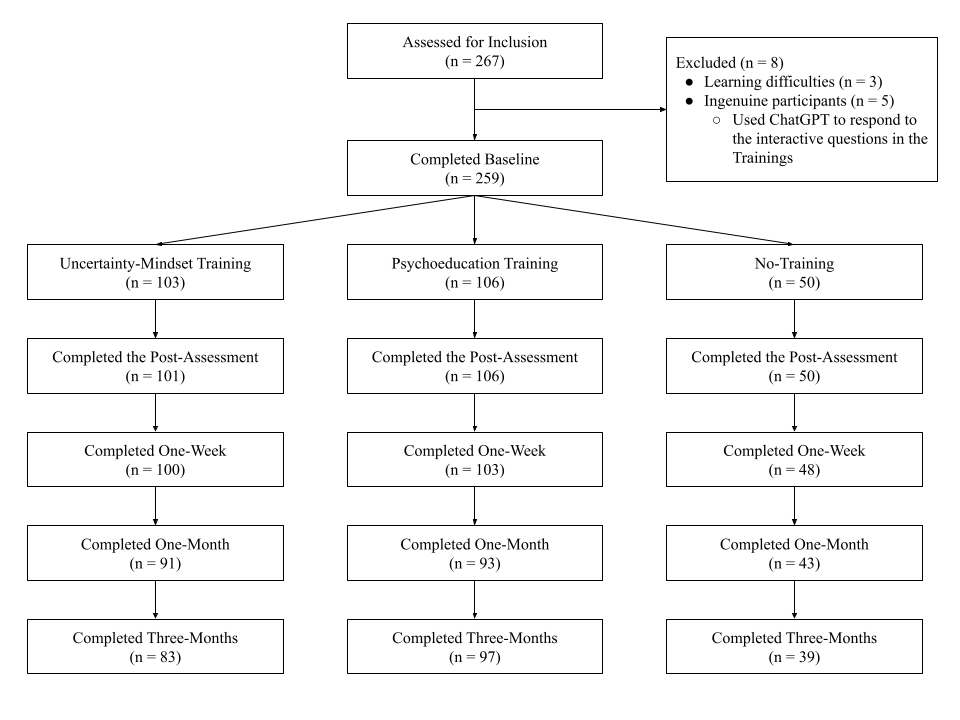


**Supplementary Specificity Analyses**

IU is associated with anxiety (b = 0.74, SE = 0.10, t = 7.80, p < .001) and depression (b = 0.57, SE = 0.09, t = 6.34, p < .001) over and above its association with negative affect. Similarly, the groups differ in the change in IU at the post-assessment (*F*(2,255) = 12.75, *p* < .001), one-week (*F*(2,251) = 10.07, *p* < .001), and one-month (*F*(2,236) = 12.23, *p* < .001) follow-ups, and for the change in anxiety (*F*(2,239) = 5.49, *p* = .005) and depression (*F*(2,239) = 4.40, *p* = .013) at one-month even when accounting for negative affect in the same model. Furthermore, within the Uncertainty-Mindset group, the effect of Time on IU (post-assessment: *β* = -0.60, CI [-0.75, -0.44], *p* < .001; one-week: *β* = -0.46, CI [-0.61, -0.30], *p* < .001; one-month: *β* = -0.47, CI [-0.64, -0.30], *p* < .001), anxiety (one-month: *β* = -0.31, CI [-0.48, -0.15], *p* < .001), and depression (one-month: *β* = -0.39, CI [-0.57, -0.21], *p* < .001) all remain significant when including negative affect as a covariate.

**Behavioural Task – Decision Making Under Uncertainty**

While IU is predominantly assessed with self-report measures (i.e., cognitive IU), studies often fail to leverage decision-under-uncertainty behavioural tasks (i.e., behavioural IU)^1^. These tasks can capture specific actions performed when facing uncertainty, such as information seeking and avoidance, which are known to contribute to the poor mental health of those with high IU^2,3^.

As such, the present study sought to investigate the association between self-reported and behavioural IU, and whether behavioural IU can be improved through a single-session online training. While these hypotheses were pre-registered (https://osf.io/fztqr), the results are reported in the supplementary materials document given that the behavioural task used was not sensitive in the current sample and its’ interpretability was limited. Specifically, levels of sampling (an indication of information seeking in our task) were very low across our participants (substantially lower than that of the original study from which the task was derived^4^). Indeed, almost one third of participants (29.6%) never sought any information about either option’s reward contingencies prior to making any decision, and instead were just choosing blindly, suggesting the task may not have been readily understood by participants. Additionally, the vast majority (74.1%) of those who did seek information, did so less than twice prior to making each decision. Given that our behavioural task likely did not accurately capture behavioural IU as intended, these results should be approached with caution and require replication in future research.

**Methods**

Behavioural IU was assessed using a decision-under-uncertainty paradigm that measures information seeking^4^. In this task (Figure S2), participants are presented with 10 dual-option decision trials. On each trial, participants see two rectangles that each correspond to one of two circles above them. Participants were told that they will win or lose points when they select a circle in each round. The aim of the game was to earn as many points as possible, with points being translated to a monetary bonus of up to £5 (median = £1.5).

**Supplementary Figure 2: Screen progression of each trial of the behavioural task**

On each trial, the number of points to be won and the probability of each choice leading to a win/loss was unknown (i.e., uncertain). Participants could reduce their uncertainty about each option’s reward contingencies through sampling. They did this by clicking on the rectangles associated with each circle option. They could sample outcomes as many times as they liked, up to a maximum of 30 samples at which point the task moved on to the next round. By sampling, participants learned how many points they would have won if they had clicked on the corresponding circle at that point. Points shown when sampling did not contribute to the participant’s total score. When they were ready to make a choice that contributed to their score, participants clicked on one of the two circles. Once participants chose a circle, they were told the number of points they received for that round and their cumulative point totals. Behavioural IU was operationalised as the number of times participants sampled prior to making a decision in the 10 trials (i.e., a higher score indicates greater information seeking and thus a higher behavioural IU).

***Data Analyses***

To investigate the relationship between cognitive and behavioural IU, a mixed effects model was run with participant ID as the random factor, and trial number and cognitive IU as fixed factors. A mixed effects model was also used to investigate the fixed and interacting effects of Group and Time on behavioural IU (sampling across the 10 trials was averaged to give one behavioural IU score per participant for each of the baseline and post-assessments), with participant ID included as the random effect. Twelve participants who did not understand the behavioural task, evidenced by them never making a choice in any of the 10 trials at baseline, were excluded from the analyses.

**Results and Discussion**

***Relationship Between Cognitive and Behavioural Intolerance of Uncertainty at Baseline***

Both cognitive IU and trial number had main effects on sampling in the behavioural task at baseline (Table S5), such that sampling was lower for those with higher cognitive IU and at later trials. Additionally, cognitive IU significantly interacted with trial number (Table S5; Figure S3). More specifically, there was a greater decrease in sampling as the task progressed for those with lower cognitive IU, whereas sampling levels were low across the entire task for those with higher cognitive IU. However, Bayesian analyses did not find evidence for the main or interacting effects found in the frequentist analyses (Table S5), and as such, the following conclusions are tentative.

Individuals high in IU may have exhibited low levels of sampling across the entire task because they experience uncertainty as highly aversive and thus desire to avoid remaining in the state of uncertainty created by the task (i.e., complete the task as quickly as possible). Whereas participants with lower IU may have exhibited greater sampling to learn about the options’ reward contingencies, because they are better able to handle the uncertainty and thus not having the urge to avoid it. This interpretation aligns with the results of a study in which participants were first presented with a risky but low value reward option (i.e., 50% chance of winning $0.04) which they can accept and be immediately told if they had won the reward, or they can choose to wait a varying amount of time for a safer and more valuable reward option (i.e., 70% chance of $0.06)^5^. In this study, those with a higher IU were less likely to wait for the better second option, indicative of a desire to reduce the amount of time spent experiencing uncertainty because of its aversiveness^5^. The decrease in sampling as the trials progressed for those with low IU may be explained by individuals getting bored or fatigued, and thus becoming increasingly motivated to complete the study quickly. While these results provide some preliminary evidence for greater avoidance in those with high IU, whether this avoidance is driven by negative emotional reactions to uncertainty is speculative and needs to be empirically tested in the future.

**Supplementary Table 5: Mixed effects model investigating the effects of cognitive intolerance of uncertainty and trial number on behavioural intolerance of uncertainty at the baseline assessment**

|  | **Behavioural Intolerance of Uncertainty** | | | |
| --- | --- | --- | --- | --- |
|  | *F* | *df* | *p* | *BF_10_* |
| Cognitive intolerance of uncertainty | 5.61 | (1,245) | .019 | 0.021* |
| Trial number | 11.04 | (9,2205) | <.001 | <.001* |
| Cognitive intolerance of uncertainty x Trial number | 8.41 | (9,2205) | <.001 | <.001* |

Cognitive intolerance of uncertainty: self-reported on the 12-item Intolerance of Uncertainty Scale at baseline^6^. Trial number: Trials 1 through 10. Behavioural intolerance of uncertainty: the number of times participants sampled prior to making a decision in each trial at baseline. The significance threshold utilised for the frequentist analyses is *p* < .025. *BF_10_* is the Bayes Factor ratio of the evidence for the alternative hypothesis over the null hypothesis (H_1_:H_0_). Bayes factors are interpreted as anecdotal (1-3), moderate (3-10), strong (10-30), very strong (30-100), and extreme (>100) evidence for H_1_, and anecdotal (*BF*_10_ = 0.33-1), moderate (0.33-0.10), strong (0.10-0.03), very strong (0.03-0.01), and extreme (<0.01) evidence for H_0_^7^. * indicates where there is a discrepancy between the outcomes of the frequentist and Bayesian analyses.

**Supplementary Figure 3: Effects of cognitive intolerance of uncertainty and trial number on behavioural intolerance of uncertainty at the baseline assessment**


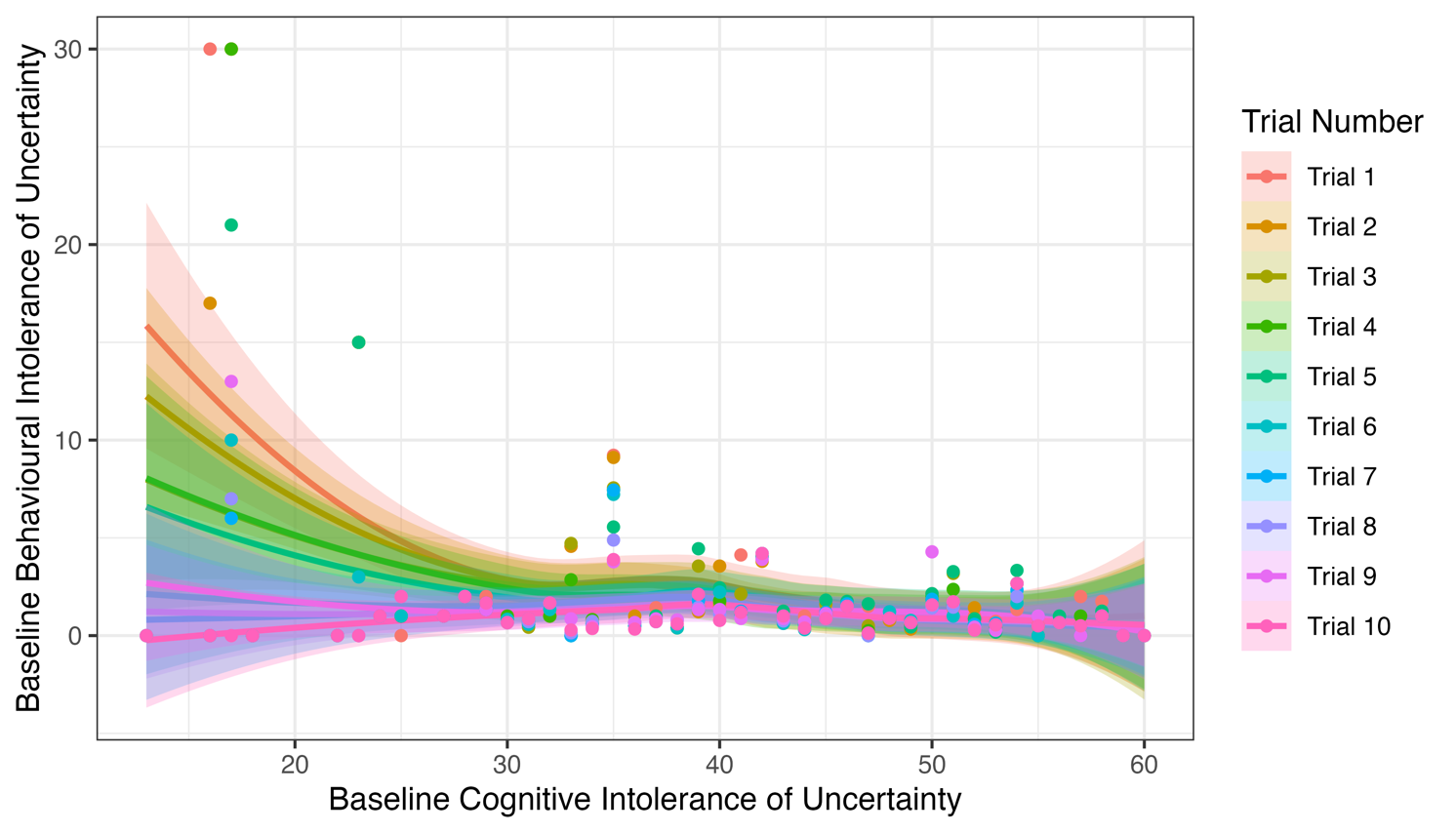


Cognitive intolerance of uncertainty was measured with the 12-item Intolerance of Uncertainty Scale^6^. Behavioural intolerance of uncertainty is operationalised as the number of times participants sampled prior to making a decision in each of the 10 trials (i.e., a higher score indicates greater information seeking and thus a higher behavioural IU). Each point represents the average of the number of times participants with the same cognitive intolerance of uncertainty score sampled on a respective trial. The shaded area around the trendlines represent the 95% confidence intervals.

***Changes in Behavioural Intolerance of Uncertainty***

Sampling in the behavioural task decreased from the baseline to the post-assessment, as indicated by a significant effect of Time (Table S6; Figure S4). Neither the effect of Group nor the Group by Time interaction were significant, suggesting that sampling was not differentially impacted by the Uncertainty-Mindset Training.

This baseline to post-assessment reduction in sampling was likely due to practice effects or fatigue, rather than actual changes in behavioural IU, given that the sampling reductions were experienced independent of group. The result that the Uncertainty-Mindset Training differentially impacted cognitive but not behavioural IU compared to the control groups at post-training is consistent with Deschenes et al.’s (2010)^8^ experimental manipulation study. While providing participants with information about how uncertainty can help (i.e., uncertainty is positive) or hinder (i.e., uncertainty is negative) problem solving resulted in different self-reported scores on the Intolerance of Uncertainty Scale, it did not result in different levels of sampling in their behavioural task at the post-assessment^8^. While it may be the case that the Uncertainty-Mindset Training is simply not effective in improving behavioural IU, it is also possible that behavioural responses to uncertainty may not be immediately amenable, rather they may require more time and experience to shift. Therefore, future studies should continue to assess behavioural IU beyond the immediate post-assessment.

**Supplementary Table 6: Mixed effects model investigating the effects of group and time on behavioural intolerance of uncertainty**

|  | **Behavioural Intolerance of Uncertainty** | | | |
| --- | --- | --- | --- | --- |
|  | *F* | *df* | *p* | *BF_10_* |
| Group | 0.00 | (2,245) | .997 | 0.002 |
| Time | 5.81 | (1,241) | .017 | 1.46 |
| Group x Time | 1.96 | (2,242) | .143 | 0.011 |

Group: No-Training vs. Psychoeducation vs. Uncertainty-Mindset Training. Time: Baseline and post-assessment. The significance threshold utilised for the frequentist analyses is *p* < .025. *BF_10_* is the Bayes Factor ratio of the evidence for the alternative hypothesis over the null hypothesis (H_1_:H_0_). Bayes factors are interpreted as anecdotal (1-3), moderate (3-10), strong (10-30), very strong (30-100), and extreme (>100) evidence for H_1_, and anecdotal (*BF*_10_ = 0.33-1), moderate (0.33-0.10), strong (0.10-0.03), very strong (0.03-0.01), and extreme (<0.01) evidence for H_0_^7^.

**Supplementary Figure 4: Training effects on behavioural intolerance of uncertainty**


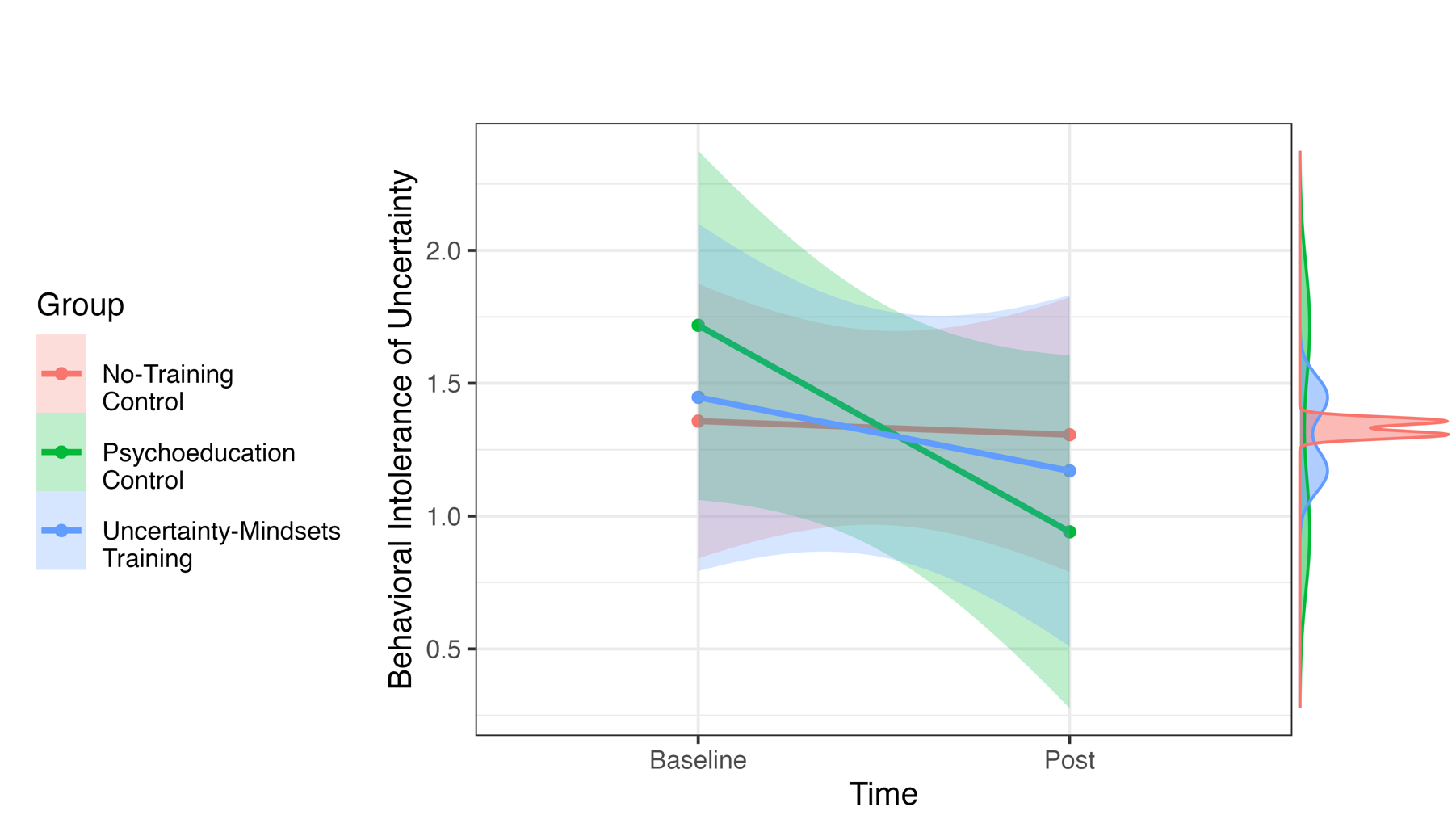
**
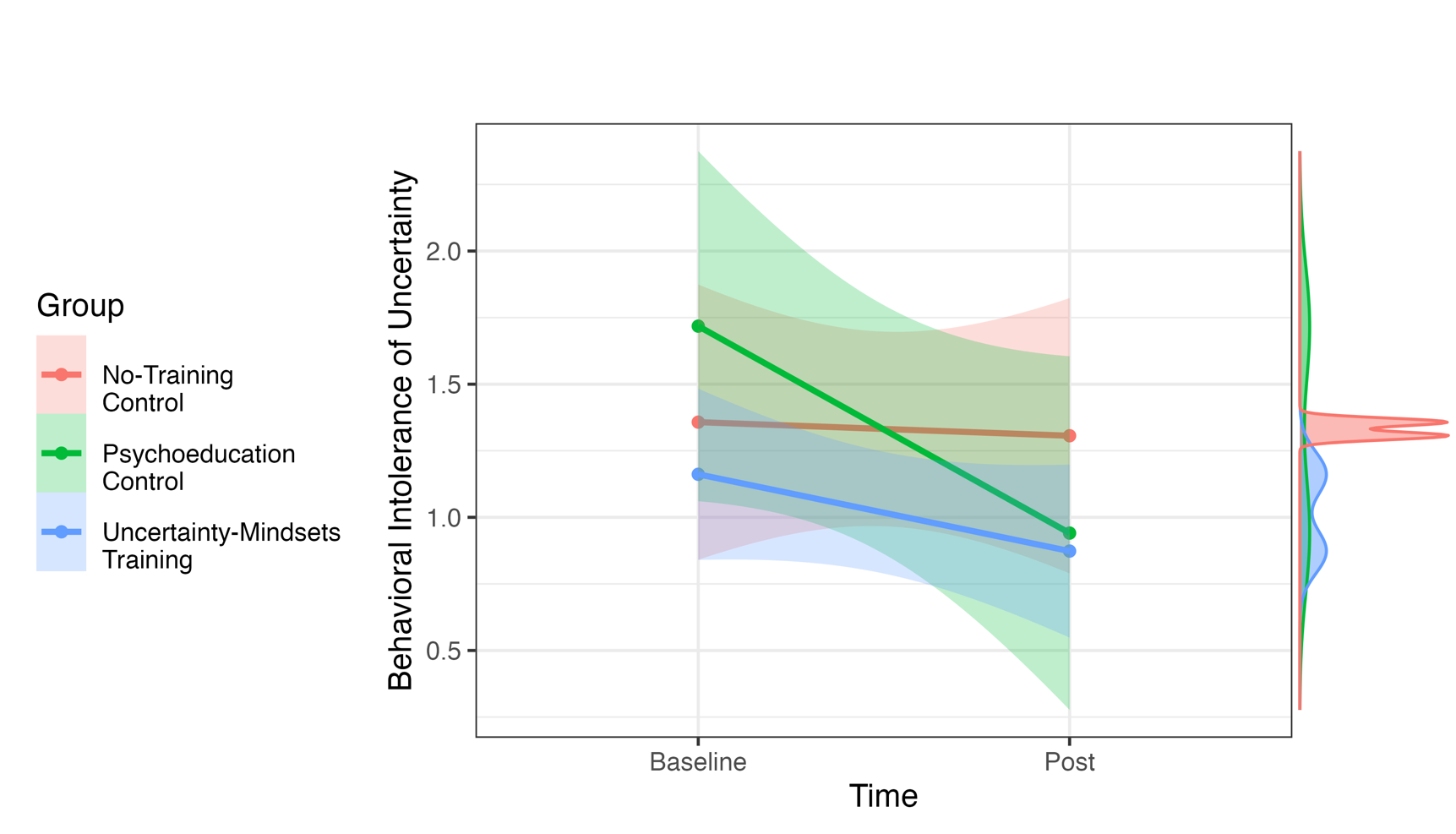
**

Behavioural intolerance of uncertainty is operationalised as the average number of times participants sampled prior to making a decision across all 10 trials (i.e., a higher score indicates greater information seeking and thus a higher behavioural IU).

**Behavioural Task – References**

1. Osmanağaoğlu, N., Creswell, C. & Dodd, H. F. Development of a behavioural measure of Intolerance of Uncertainty in preadolescent children: Adaptation of the beads task. *Journal of Behavior Therapy and Experimental Psychiatry* **72**, 101654 (2021).

2. Bottesi, G., Marino, C., Vieno, A., Ghisi, M. & Spada, M. M. Psychological distress in the context of the COVID-19 pandemic: the joint contribution of intolerance of uncertainty and cyberchondria. *Psychology & Health* **37**, 1396–1413 (2022).

3. Kurita, K., Garon, E. B., Stanton, A. L. & Meyerowitz, B. E. Uncertainty and psychological adjustment in patients with lung cancer: Uncertainty predicts psychological adjustment. *Psycho-Oncology* **22**, 1396–1401 (2013).

4. van den Bos, W. & Hertwig, R. Adolescents display distinctive tolerance to ambiguity and to uncertainty during risky decision making. *Sci Rep* **7**, 40962 (2017).

5. Luhmann, C. C., Ishida, K. & Hajcak, G. Intolerance of Uncertainty and Decisions About Delayed, Probabilistic Rewards. *Behavior Therapy* **42**, 378–386 (2011).

6. Carleton, R. N., Norton, M. A. P. J. & Asmundson, G. J. G. Fearing the unknown: A short version of the Intolerance of Uncertainty Scale. *Journal of Anxiety Disorders* **21**, 105–117 (2007).

7. Andraszewicz, S. *et al.* An Introduction to Bayesian Hypothesis Testing for Management Research.

8. Deschenes, S. S., Dugas, M. J., Radomsky, A. S. & Buhr, K. Experimental Manipulation of Beliefs about Uncertainty: Effects on Interpretive Processing and Access to Threat Schemata. *Journal of Experimental Psychopathology* **1**, jep.008510 (2010).
